# *Flueggea acicularis* (Phyllanthaceae), a narrow endemic species rediscovered in central China

**DOI:** 10.3897/phytokeys.172.57217

**Published:** 2021-02-10

**Authors:** Songzhi Xu, Qiliang Gan, Lianzhong Fu, Mingxi Jiang, Zhenyu Li

**Affiliations:** 1 School of Life Science, Nantong University, 226019, Nantong, China Nantong University Nantong China; 2 Zhuxi Qiliang Biological Institute, Zhuxi 442300, Hubei, China Zhuxi Qiliang Biological Institute Zhuxi China; 3 State Key Laboratory of Systematic and Evolutionary Botany, Institute of Botany, Chinese Academy of Sciences, 100093, Beijing, China Institute of Botany, Chinese Academy of Sciences Beijing China; 4 Key Laboratory of Aquatic Botany and Watershed Ecology, Wuhan Institute of Botany, Chinese Academy of Science, Wuhan 430074, China Wuhan Institute of Botany, Chinese Academy of Science Wuhan China

**Keywords:** *Flueggea acicularis*, morphology, rediscovery, taxonomy, Three Gorges Area

## Abstract

*Flueggea acicularis* (Phyllanthaceae) is endemic to the karst region of central China. Male specimens of this species were first collected in 1908. In 1989, female plants of *F. acicularis* were found for the first time, but misidentified as a new species. Throughout this period the male plants of *F. acicularis* were mismatched with female plants of other species, and male plants had not been collected since 1908. Then, in March, 2009, the authors rediscovered a wild population of *F. acicularis* consisting of both male and female plants in Wuxi county, Chongqing municipality, China. Based on field investigation and examination of specimens, we matched the correct female and male plants of this species for the first time since its initial publication a century ago. A complete and accurate morphological description, distribution, habitat and phenology of this species are also provided. Furthermore, the conservation status of *F. acicularis* is assessed as “Near Threatened” (NT) according to the IUCN Red List criteria.

## Introduction

*Flueggea* (Phyllanthaceae) consists of 16 species, widespread in tropical to warm temperate regions, along with 3 Eurasian narrowly distributed taxa that have been interpreted as relictual. An unusually obligate pollination mutualism (pollination and seed parasite) exists between Epicephala moth and Phyllanthaceae trees (Kawakita 2010, Hu and Li 2011). Hu et al. (2011) also reported that *Epicephala relictella* fed on the seeds of *F. suffruticosa*, but was not pollinating its host. Some secondary products (alkaloids, diterpenoids) that are of medicinal use have been extracted from *Flueggea* (Hu et al. 2011; Wang et al. 2010).There are four *Flueggea* species in China, and *Flueggea acicularis* is endemic to the karst region of central China. *F. acicularis* is extremely similar to *F. tinctoria* in morphology (Webster 1984). However, the geographical disjunction between the Chinese *F. acicularis* and *F. tinctoria* in the Iberian Peninsula is one of the most remarkable in the Euphorbiaceae, which might represent the relicts of the flora of Tethys in Tertiary period. But the taxonomic status of *F. acicularis* is somewhat in doubt.

The English botanist E.H. Wilson collected a male shrub assigned to Euphorbiaceae s.l. in the Three Gorges Area of central China in March, 1908. Hutchinson (1916) misidentified these specimens as *Flueggea leucopyra* Willd., a species producing berries. Croizat (1940) published a new species *Securinega acicularis* Croizat based on the three specimens of Wilson. Airy Shaw (1971) accepted this new species and described the morphology of fruits as “a shallowly 3-lobed, depressed-globose, dehiscent capsule, 6–7mm in diam.”, but no specimens documenting the fruit were cited. Webster (1984) used a broad generic concept in the taxonomic revision of *Flueggea* and combined *Securinega acicularis* Croizat into *Flueggea acicularis* (Croizat) Webster, but indicated that pistillate flowers and the fruit were not seen. Li (1994) and Li and Gilbert (2008) described this species with “Female flowers: pedicels ca. 3mm; sepals 5,……Berry globose, 6–7 mm in diam, 3-locular”. Thus, up to this point, the taxonomic status of *F. acicularis* and the morphological description of the staminate plant have not been doubted in academia, but the morphological characters of pistillate flowers and capsules have been confused throughout this period.

After a careful examination of the specimens stored under the name of *Flueggea acicularis* in main herbaria of China, we found that 25 sheets representing ten gatherings collected from the Jinshajiang valley flanking northwestern Yunnan province and southwestern Sichuan province had been misidentified. The whole plants of these specimens are entirely glabrous and bear flowers with 5 sepals, clearly differing from *F. acicularis* which has hirtellous young branchlets and has 6 sepals in a flower. These misidentified specimens belong to *Flueggea virosa* (Roxb. ex Willd.) Voigt, *F. suffruticosa* (Pall.) Baill. and *F. leucopyra* Willd. Besides, a most recent study of Huang et al. (2020) claimed that diterpenoids have been extracted from the aerial parts of *F. acicularis* collected in Yunnan. Given that the locality of collection is in Yunnan, it seems that the materials used in their study should be *F. leucopyra*, instead of *F. acicularis*.

In August, 1989, three botanists (Mingxi Jiang, Zongqiang Xie and Jinsheng He) collected specimens of the female plants of *F. acicularis* for the first time, in Luyoudong, Wushan county, during the vegetation investigation in the Three Gorges Area. But the specimens were not identified, because of their lack of flowers and fruits. In May, 1990, Zongqiang Xie et al. collected specimens of *F. acicularis* with capsules in Bawuxia located in the midstream of Daning river. However, since most floras in China still confused *F. acicularis* with *F. leucopyra* (Fu, 1979), or considered *F. acicularis* to have berries (Li 1994), the real female specimens of *F. acicularis* with capsules were misidentified as a new species under a nomen nudum “*Securinega wuxiensis*” (Chen et al. 1994). Thus, the male plants of *F. acicularis* remained paired with female plants of other species, and the true female plants of *F. acicularis* were considered as another species.

In April, 2009, during a field trip to Wuxi county, Chongqing municipality, the authors accidentally discovered a population consisting of shrubs of Phyllanthaceae in a remote area called Jingzhuba. The place is located in the limestone canyon of the headwaters of the Daning river in the northeast of Wuxi county. We collected specimens of both male and female plants containing staminate and pistillate flowers and capsules from the same population. After a careful morphological investigation, we confirmed that this species is *F. acicularis*. Based on an intensive study of the male and female plants from the same population, the male plant of *F. acicularis* has finally been matched with the correct female plant of the same species.

## Materials

Specimens were collected and photographs were taken in the field of Badong county, Hubei, Wushan county, and Wuxi county, Chongqing municipality, Central China in 1989, 1990, 1997 and 2009. Specimens from the main herbaria of China (PE, KUN, IBK, IBSC) and some digital specimen databases (CVH, A, MO, US, K, GH, JSTOR Global Plants) were checked. The morphology of the species was observed and measured based on living plants, dry specimens and preserved materials. All morphological characters were measured with dissecting microscopes and were described using the terminology presented in Harris and Harris (1994).

## Taxonomy

### Flueggea acicularis

Taxon classificationPlantaeMalpighialesPhyllanthaceae

(Croizat) Webster in Allertonia 3(4): 304. 1984.

DB864E66-CAA7-52B1-8D27-5B3CBE305192

Securinega acicularis Croizat in Journ. Arn. Arb. 21(4):491. 1940.
Flueggea leucopyra auct. *non* Willd.: Hutch. in Sargent, Pl. Wilson. 2:520. 1916.

#### Types.

China: Hubei (Hupeh) province, Badong (Patung Hsien) county, bush 2–6 feet, cliffs and rocky places, alt. 30–304 m, 24 March 1908, E. H. Wilson 3336 (**holotype**, A, A00048778; **isotypes**, A, GH, K, MO, US); l.c.3335 (paratypes, A, US, K); Chongqing municipality (former eastern Sichuan), Wushan county, Wu Gorge (Wu Xia or Wushan Gorge), bush 3–4 feet, March 1908, E. H. Wilson 3344 (paratypes, A, K). (All male specimens, photos, PE!).

#### Additional specimens.

**Figure 1.**
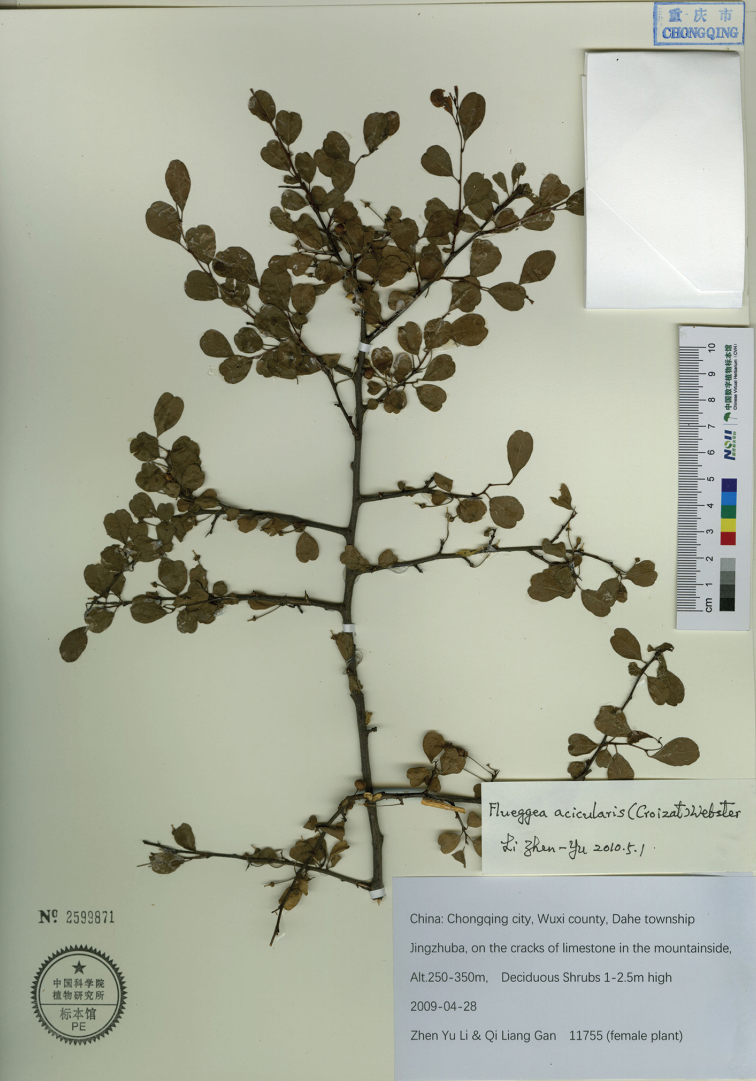
*Flueggea acicularis* (Croizat) Webster. (fruiting specimen, Z. Y. Li & Q. L. Gan 11755, PE).

China: Chongqing municipality, Wuxi county, Jingzhuba, 2009-04-28, Zhen Yu Li & Qi Liang Gan 11751 (female plant, 2 sheets, PE!); 1.c.11755 (female plant, 2 sheets, PE!) (Fig. 1); 1.c. 11758 (male plant, 2 sheets, PE!); and 11759 (male plant, 2 sheets, PE!).

#### Description.

**Figure 2.**
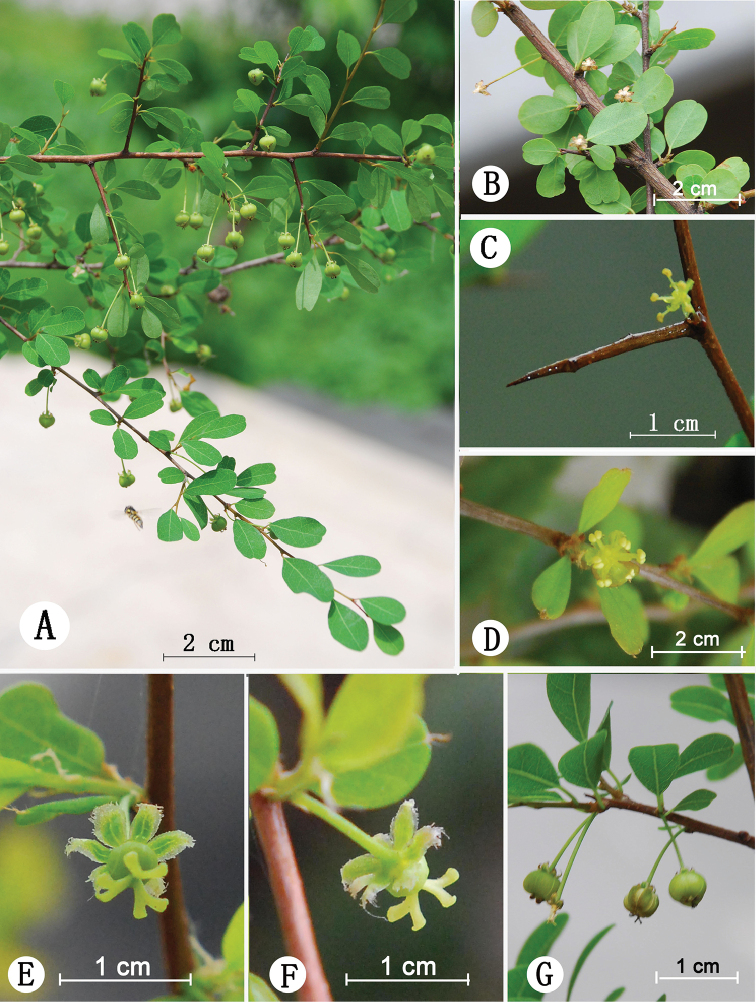
Living plants of *Flueggea acicularis* (Croizat) Webster **A** fruiting branches **B** fruit remnants after dehiscing **C** branches and spines **D** staminate flower **E, F** pistillate flower **G** capsules (note epicarp dehiscence lines in center fruit).

**Figure 3.**
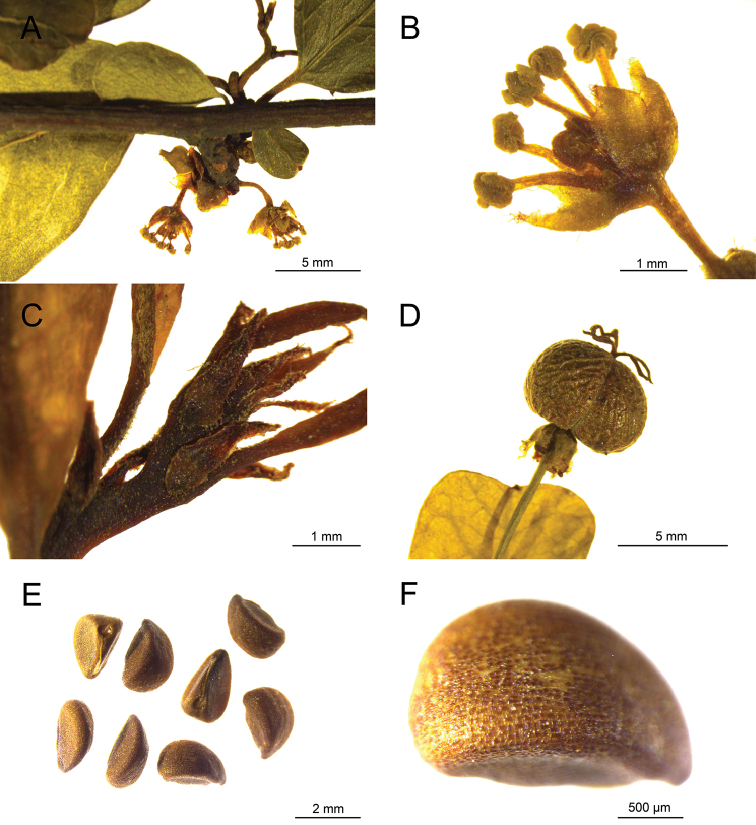
Herbarium collections of *Flueggea acicularis* (Croizat) Webster **A** staminate inflorescence **B** staminate flower **C** young leaves and stipules **D** capsule **E** seeds **F** seed (back view) (**A, B** Z. Y. Li & Q. L. Gan 11758, PE; **C, D, F** Z. Y. Li & Q. L. Gan 11751, PE).

Shrubs, 1–2.5 m high, deciduous. Stems many branched, branches alternate, diverging into an obtuse angle and arranged in two rows, usually with lateral and terminal spines; branchlets of current year’s growth copiously hirtellous, angulate, yellow-brown, gray-brown, glabrate and nitid when old. Leaves alternate, distichous, or 1–3 fascicled on upper part of a branchlet; stipules reddish-brown, lanceolate, 0.7–2 mm long, margins ciliate, apex caudate-acuminate, persistent; petioles 1–5 mm long, copiously hirtellous when young, later glabrate; leaf blade oblong-obovate to obovate, 0.5–2 cm long, 0.4–1.5 cm wide, thickly papery in texture, glabrous except young midrib base, base cuneate, margin entire, flat or narrowly revolute when dry, apex retuse, obtuse or rounded, mucronulate, adaxially green, slightly lustrous, abaxially pale green, midrib extending to apex, lateral veins 4–6 on each side of midrib, alternate, rarely opposite, connected near margin, midrib and lateral veins slightly raised on both surfaces, veinlets reticulate. Plants dioecious. Male inflorescence with solitary staminate flower, axillary; bracts inconspicuous; pedicel 2–5(–7) mm long; sepals 6, ovate-oblong or elliptic, 1.2–1.6 mm long, 0.4–1.3 mm wide, apex rounded, margin erose or fimbriate, membranous, imbricate, slightly recurved, disk glands 6, angled, coherent, 0.4–0.5 mm across; petals absent; stamens 6, free, filaments green-yellow, 1.5–2mm long, anthers yellow, 0.6–1 mm long, theca parallel, longitudinally dehiscent, anther connectives not protruding; pistillode with 3(rarely 2) branches fused to the middle or beyond, the fused portion 0.8–1.5 mm high, the sharply recurved tips 0.4–1mm long. Female inflorescence 1-rarely 2-flowered, arising in leaf axil; bracts inconspicuous. Pistillate flower: sepals 6, ovate-oblong or oblong, 1.5–1.8 mm long, 0.6–0.7 mm wide, apex acute, margin membranous, fimbriate; persistent and reflexed after anthesis; pedicels 5–8 (–10) mm long that elongate to 16 mm in fruit, pendulous; disk angular, 0.8–1.3 mm across; petals absent; ovary globose, 3-locules, each with 2 ovules; styles 3, patent, flattened, each 2-lobed to middle, stigmas rounded at apex, styles and stigmas yellow-green. Capsules oblate, somewhat triangular in cross section, glabrous, ca. 5 mm in diam., green, brown when dry with persistent and nigrescent styles and stigmas, dehiscing through the locules when mature, valves and seeds falling off after dehiscing, columella persistent, 2–2.2 mm long. Seeds ovoid-trigonous, brown, 2.3–2.8 mm long, 1.6–1.7 mm broad at back, ca. 1.5 mm thick, glabrous, with raised pigmented minute rectangular testa cells (Figs 2, 3).

#### Distribution.

China: Hubei province (Badong county); Chongqing municipality (Wushan county and Wuxi county), alt. (formerly 30–300 m) 175–300 m.

#### Phenology.

Flowering and fruiting from April to May.

#### Chinese name.

Mao bai fan shu (hairy flueggea) (Li 1994), refers to the copiously hirtellous.

#### Local name.

Yang ci.

#### Habitat and ecology.

Three Gorges reservoir area is located in a subtropical region. The annual average rainfall is 1000–1400 mm, mostly in July and August. The annual average temperature is 18.4 °C (average temperature in January: 7.1 °C; average temperature in July: 29.3 °C), and the extreme maximum temperature is up to 44 °C. The relative humidity is 60%–80%. The photoperiod in this area is short, affected by the canyon landform and the foggy environment.

**The population of Badong.** In March, 1908, the type specimen was collected from Badong county, in cliffs and rocky places in the bank of Yangtze river at altitudes from 30 to 300 m, without detailed location. In May, 1997, Mingxi Jiang found ca. 2,000 individuals of *F. acicularis* in Mazongshan village (31°2'36"N, 110°9'59"E), Badong county. It grows on a slope located in the bank of Yangtze River, at alt. 100–300 m. The main companion species include shrubs: *Maytenus variabilis* (Hemsl.) C. Y. Cheng, *Viburnum utile* Hemsl., Vitex regundo var. heterophylla (Franch.) Rehd.; woody liana: *Bauhinia brachycarpa* Wall. ex Benth.

**The population of Wushan county.** In 1908, Wilson collected specimens of *F. acicularis* here, but without detailed location. In August, 1989, Mingxi Jiang et al. found more than 10,000 individuals of *F. acicularis* in Luyoudong (30°36'24"N, 108°25'30"E), Wushan county. It grows on limestone on the banks of the Yangtze River, at alt. 100–300 m. The main companion species were similar to Mazongshan population. In May, 1990, Zongqiang Xie and Mingxi Jiang et al. found another monodominant community of *F. acicularis* in Bawuxia in the midstream of Daning river, Wushan county, at alt. 50–250m. The main companion species include shrubs: *Euonymus alatus* (Thunb.) Sieb., *Lespedeza formosa* (Vog.) Koehne, *Sageretia thea* (Osbeck) Johnst., Vitex regundo var. heterophylla (Franch.) Rehd., *Zanthoxylum armatum* DC. etc; herbs: *Arthraxon lanceolatus* (Roxb.) Hochst., *Phyllanthus urinaria* L.; woody liana: *Clematis armandii* Franch., *Millettia reticulata* Benth.; herbaceous vines: *Cayratia japonica* Gagnep. and *Dioscorea oppositifolia* L. (Xie and Jiang 1995).

**The population of Wuxi county.** This population was located in Jingzhuba, off headwaters of the Daning river, Dahe township, Wuxi county (31°21'40"N, 109°24'34"E). It grows in the cracks of limestone on south slopes, at elevations of ca. 250–350 m. The rock surface is mostly exposed and the soil layer is extremely thin. The vegetation consists of open shrubland with heights lower than 5 m and the main companion species include shrubs: Boehmeria clidemioides var. diffusa (Wedd.) Hand.-Mazz., *Broussonetia kazincki* Siebold, *Buddleja davidii* Franch., Cotinus coggyria var. pubescens Engl., *Debregeasia orientalis* C.J. Chen, *Flueggea acicularis* (Croizat) Webster, *Itea illcifolia* Oliv., *Lespedeza floribunda* Bunge and *L. formosa* (Vog.) Koehne; woody lianas: *Ampelopsis aconitifolia* Bunge, *Holboellia fargesii* Reaub., *Parthenocissus dalzielii* Gagnep.; herbs: *Artemisia annua* L., *A. sylvatica* Maxim., *Arthraxon lanceolatus* (Roxb.) Hochst, *Boea hygrometrica* (Bunge) R. Br., *Bothriospermum zeylanicum* (J. Jacq.) Druce, *Carex brevicuspis* C.B. Clarke, *Corydalis ophiocarpa* Hook. f. et Thoms., *Duchesnea indica* (And.) Focke, *Eriophorum comosum* Nees, *Hemistepta lyrata* (Bunge) Bunge, *Miscanthus sinensis* Anderss., *Oxalis corniculata* L., *Setaria viridis* (L.) Beauv., *Stellaria media* (L.) Cyr., *Toridis japonica* (Houtt.) DC., *Youngia heterophylla* (Hemsl.) Babc. et Stebb.; pteridophytes: *Adianthum capillus*-*veneris* L., *Hypodematium crenatum* (Forssk.) Kuhn, *Pteris vittata* L. and *Selaginella davidii* Franch.; mosses: *Conocephalum conicum* (L.) Dumort; herbaceous vines: Cayratia japonica var. pseudotrifoliata (W.T. Wang) C.L. Li and *Paederia foetida* L.. There are about 80 individuals including ca. 20 seedlings, and pistillate plants are in the majority. Since the population is close to the road, the alien weeds *Conyza canadensis* (L.) Cronq., Bidens pilosa var. radiate Sch.-Bip. and *Veronica persica* Poir. have invaded the edge of the population.

Provisional IUCN conservation assessment. In 2009, the Three Gorges water conservancy project, the largest in the world, was completed, located in Yichang city, Hubei province. The altitudes of the dam base and dam top are 4 m and 185 m respectively, and therefore we calculated the populations of Badong and Wushan of *F. acicularis* at alt. lower than 185m have been submerged according to altitude. Based on field investigations and specimens, we are confident that *F. acicularis* is distributed narrowly, and is endemic to the karst region of the Three Gorges Area in Central China. The provisional conservation status is “Near Threatened” (NT) according to the IUCN red list criteria (IUCN 2019).

## Supplementary Material

XML Treatment for Flueggea acicularis

## References

[B1] Airy Shaw HK (1971) Notes on Malesian and other Asiatic Euphorbiaceae. Kew Bulletin 25(3): 473–553. 10.2307/4103199

[B2] Chen WL Liang SY Jin YX Yang QX (1994) Plants and the complex of agriculture ecosystems in the Three Gorges Region. Science Press, Beijing, 136 pp.

[B3] Croizat L (1940) New and critical Euphorbiaceae from eastern tropical Asia. Journal of the Arnold Arboretum 21(4): 490–510. https://www.jstor.org/stable/43780966

[B4] Fu SH (1979) Flora Hubeiensis, vol. 2. Hubei People’s Publishing House, Wushan, 361–362.

[B5] Harris JG Harris MW (1994) Plant identification terminology: an illustrated glossary. Spring Lake Publishing, Payson, 188 pp 10.2307/1222694

[B6] Hu BB Li HH (2011) Advance in the study of the mutualism between epicephala moths (Lepidoptera, Gracillariidae) and Euphorbiaceae plants in china. Dong Wu Fen Lei Xue Bao 36(2): 447–457.

[B7] Hu BB Wang SX Zhang J Li HH (2011) Taxonomy and biology of two seed-parasitic gracillariid moths (Lepidoptera, Gracillariidae), with description of a new species. ZooKeys 83(83): 43–56. 10.3897/zookeys.83.783 PMC3082966 21594087

[B8] Huang D Luo XK Yin ZY Xu J Gu Q (2020) Diterpenoids from the aerial parts of *Flueggea acicularis* and their activity against RANKL-induced osteoclastogenesis. Bioorganic Chemistry 94: 103453. 10.1016/j.bioorg.2019.103453 31787342

[B9] Hutchinson J (1916) Euphorbiaceae. In: Saegent CS (Ed.) Plantae Wilsonianae 2.The University Press, Cambridge, 516–529.

[B10] IUCN (2019) Guidelines for Using the IUCN Red List Categories and Criteria. Version 14. Prepared by the Standards and Petitions Committee. http://www.iucnredlist.org/documents/RedListGuidelines.pdf

[B11] Kawakita A (2010) Evolution of obligate pollination mutualism in the tribe Phyllantheae (Phyllanthaceae). Plant Species Biology 25(1): 3–19. 10.1111/j.1442-1984.2009.00266.x

[B12] Li BT (1994) Euphorbiaceae, Phyllanthoideae. In: Li BT (Ed.) Flora Reipublicae Popularis Sinicae Tomus, vol.44. Science Press, Beijing, 1–217.

[B13] Li BT Gilbert MG (2008) *Flueggea*. In: Wu ZY Raven PH Hong DY (Eds) Flora of China, vol.11. Science Press, Beijing and Missouri Botanical Garden Press, St. Louis, 177–179.

[B14] Wang GC Wang Y Zhang XQ Li YL Yao XS Ye WC (2010) Securinega alkaloids from *flueggea leucopyra*. Chemical & Pharmaceutical Bulletin 58(3): 390–393. 10.1248/cpb.58.390 20190447

[B15] Webster GL (1984) A revision of *Flueggea* (Euphorbiaceae). Allertonia 3(4): 259–312.

[B16] Xie ZQ Jiang MX (1995) The characteristics and utilization of shrub vegetation in the limestone area of Sanxia region. Zhiwuxue Tongbao 12: 85–89.

